# 3D printed aligner materials: *In vitro* testing of biocompatibility, cytotoxicity and antimicrobial activity

**DOI:** 10.6026/973206300214890

**Published:** 2025-12-15

**Authors:** Akshaya Rajagopal, Kavitha Sree Budda, Naif Omar Binmuhana, Roma Seshagiri, Mohammad Sohail Shaik, Siddarth Bhogi

**Affiliations:** 1 Department of Orthodontist and Dentofacial Orthopedics, Mydentist Clinic, Mumbai, India; 2 Department of Orthodontics and Dentofacial Orthopedics, SIBAR Institute of Dental Sciences, Guntur, Andhra Pradesh, India; 3 Department of Preventive Dental Science, Division of Pediatric Dentistry, Ibn Sina National College for Medical Studies, Jeddah, Saudi Arabia; 4 Department of Orthodontics and Dentofacial Orthopaedics, Care Dental College, Guntur, Andhra Pradesh, India; 5 Department of Orthodontics and Dentofacial Orthopaedics, Sri Balaji Dental College & Hospital, Hyderabad, Telangana, India

**Keywords:** 3D printing clear aligners, biocompatibility, cytotoxicity, antimicrobial activity, orthodontic materials, digital dentistry

## Abstract

Three-dimensional printing provides a new perspective to produce the clear orthodontic aligners and the biocompatibility and antimicrobial property of these materials have not been fully investigated. Therefore, it is of interest to three 3D printed aligner resins against a thermoformed control using cytotoxicity and antimicrobial assays. The biocompatibility of all materials was acceptable (>70% cell viability) but Resin C had a minorly higher level of cytotoxicity. The antimicrobial activity was low except in the case of Resin B which had decreased bacterial adhesion. All in all, 3D printed aligner resins showed reasonable biocompatible properties but need to be surface modified to endow antimicrobial functionality to be employed clinically.

## Background:

The phenomenon of clear aligner therapy has utterly changed the orthodontic specialty, as it has been widely accepted by the patient and clinician because of its excellent aesthetics, comfort and oral health maintenance in comparison to the conventional fixed appliances [1]. Traditional clear aligners are mostly produced by thermoforming the polymers like polyurethane or polyethylene terephthalate glycol [2]. Nonetheless, recent digital dentistry and additive manufacturing technology development has presented three-dimensional (3D) printing as another mechanism of fabrication of orthodontic aligners [3]. Three dimensional printing presents some of the possible benefits such as increased customization, minimized wastage of materials, higher production efficiency and the chance of introducing new properties of work directly to the printed piece [4]. Several technologies of 3D printing have been modified to print aligners such as stereolithography (SLA), digital light processing (DLP), polyjet printing, and using photopolymerizable resins with a wide range of chemical structures [5]. These resins are usually composed of methacrylate monomers, oligomers, photoinitiators and other additives, which affect mechanical properties, optical properties and biological performance [6]. Even though the 3D printed aligners have found increasing clinical use, their biological safety and antimicrobial capability have not been thoroughly investigated and it is an urgent research area [7]. Orthodontic devices are constantly in contact with soft tissues of the mouth, saliva and the multifaceted oral microbiome and therefore, demanding strictness in evaluating biocompatibility to avoid the adverse effects which may arise such as inflammatory reactions, allergic reactions or even cytotoxicity [8, 9]. Also, the oral cavity consists of a wide range of microbial habitats and orthodontic devices are possible bacteria colonization and biofilm formation [10]. Certain pathogenic bacteria like Streptococcus mutans and periodontal pathogens like Porphyromonas gingivalis may also accumulate in the environment and lead to enamel demineralization, gingivitis and periodontitis in orthodontics [11]. As a result, antimicrobial-containing materials or materials with lower adhesive power in microbes may have clinical implications as these reduce the levels of plaque and related problems [12]. Recent studies have explored mechanical properties, dimensional accuracy and force delivery attributes of 3D printed aligners [13], but little has been done to thoroughly evaluate the biological applications of 3D printed aligners comparing various formulations of resin. The literature on cytotoxicity studies in the past has shown inconsistent results based on the resin formulations, post-processing regimens and assay procedures [14]. Also, the antimicrobial qualities of 3D printed orthodontic materials are also discussed with little attention in the literature, which is also a significant gap in knowledge [15]. Therefore, it is of interest to assess and compare of the biocompatibility and cytotoxicity of three commercially available 3D printed aligner resins with traditional thermoformed aligner material in terms of antimicrobial activity.

## Materials and Methods:

## Study design and materials selection:

This comparative *in vitro* experimental study evaluated four orthodontic aligner materials: three 3D printed photopolymerizable resins and one conventional thermoformed material.

## The tested materials were:

[1] Resin A: Methacrylate-based photopolymer resin

[2] Resin B: Urethane dimethacrylate-based resin

[3] Resin C: Hybrid composite photopolymer

[4] Control: Conventional polyurethane thermoformed material

[5] Specimen Preparation

For 3D printed materials, rectangular specimens (10 x 10 x 1 mm for cytotoxicity testing; 15 x 15 x 1 mm for antimicrobial testing) were designed using CAD software (Fusion 360, Autodesk) and printed using respective manufacturers' recommended 3D printers and protocols. Resin A specimens were printed using Form 3B printer (Formlabs) with 50 µm layer thickness. Resin B specimens were printed using Tera Harz Cure printer (Graphy) with 100 µm layer thickness. Resin C specimens were printed using NextDent 5100 printer (3D Systems) with 50 µm layer thickness. All 3D printed specimens underwent standardized post-processing: washing in 95% isopropanol for 10 minutes with ultrasonic agitation, air drying and post-curing according to manufacturers' specifications using appropriate curing units. Control specimens were thermoformed from 0.75 mm thick polyurethane sheets using a vacuum forming machine (Biostar, Scheu Dental) and cut to specified dimensions. All specimens were sterilized using ethylene oxide gas sterilization (12 hours exposure, 48 hours aeration) prior to biological testing. Twenty specimens per material were prepared for each testing protocol (total n=320).

## Cytotoxicity assessment:

## Cell culture:

The culture of human gingival fibroblasts (HGF-1, ATCC CRL-2014) was performed under 37°C in 5% CO_2_ humidified atmosphere in Dulbecco's Modified Eagle Medium (DMEM) with 10% fetal bovine serum, 100 U/ml Penicillin and 100 µg/ml Streptomycin. Experiments were conducted on the cell between passages 4-8.

## Extraction protocol:

Towards ISO a 10993-12 guideline, material extracts were made. Samples were placed in DMEM, in a ratio of 3 cm 2/ml (6 specimens on each material and extraction time) and exposed to 37°C. Cell exposure was preceded by the collection of extraction media followed by filtration using 0.22 µm filters.

## MTT viability assay:

HGFs were seeded on 96-well plate at a density of 1x10^4^ cells/well and allowed to incubate after 24 hours. The use of culture medium was substituted by material extracts (200 µl/well, n=8 replicates of each material at each time point) and incubated at respective time points. The MTT reagent (3 -(4,5-dimethylthiazol-2-yl)-2, 5-diphenyltetrazolium bromide, 5 mg/ml), 20 5l/well was added and 4 hours of incubation was taken. Formazan crystals were dissolved in dimethyl sulfoxide (150 µl/well) and the absorbance of the crystals was recorded at 570 nm in microplate reader (BioTek Synergy H1). The percentage of cell viability with respect to negative control (cells in fresh medium) was calculated.

## LDH cytotoxicity assay:

The release of lactate dehydrogenase was detected using commercial LDH Cytotoxicity Detection Kit (Roche Applied science) according to the instruction of the manufacturer. A 50 µl of culture supernatants was transferred to fresh 96-well plates, reaction mixture (50 µl) was added to them and they were placed under light protection and incubated 30 minutes. The measurements of absorbance were taken at 490 nm. The release of LDH was indicated in percentage of maximum release (positive control: cells with 1% Triton X-100).

## Cell morphology evaluation:

HGFs treated with 72-hour extracts were fixed with 4% paraformaldehyde and permeabilized with 0.1% Triton X-100 and stained using fluorescent phalloidin (cytoskeleton) and DAPI (nuclei). Fluorescence microscopy (Nikon Eclipse Ti_2_) was used to examine cells and morphology was qualitatively evaluated.

## Tests of antimicrobial activity:

## Microbial Strains:

Three oral microorganisms were used, including Streptococcus mutans (ATCC 25175), Porphyromonas gingivalis (ATCC 33277) and Candida albicans (ATCC 10231). The bacterial cultures were grown in brain heart infusion (BHI) broth; *C. albicans* in Sabouraud dextrose broth. The *P. gingivalis* was cultured anaerobically (85% N_2_, 10% H_2_, 5% CO_2_).

## Agar diffusion method:

The agar plates with standardized microbial suspensions (1x10^6^ CFU/ml) were inoculated with sterile material specimens. Plates were inoculated (*S. mutans* and *C. albicans*: 37°C, 24 hours, aerobic; *P. gingivalis*: 37°C, 48 hours, anaerobic). Digital calipers were used to measure the inhibition zones in millimeters (n=6 specimens of each material of each microorganism).

## Microbial adhesion assay:

Immersion of specimens in microbial suspensions 1x10^7^ CFU/ml in each broths respectively and incubation of specimens under proper conditions 24 hours. Cells that are non-adherent were isolated through gentle washing using sterile phosphate-buffered saline. Sonication (40 kHz, 5 minutes) and vortexing in sterile saline detached adherent microorganisms. Serial dilutions were put on suitable agar media and the colony-forming units were counted after incubation (n=8 specimens per material per microorganism).

## Surface characterization:

## Scanning electron Microscopy:

To analyze surface topography, representative samples (n=3:1 material) were gold-sputtered and the samples were tested with the help of SEM (JEOL JSM-6390LV) at acceleration voltage of 15 kV and magnifications of 500x, 1000x and 5000x.

## Contact angle measurement:

The assessed method of surface wettability was sessile drop method goniometer (DataPhysics OCA 15EC). Liquid drops of deionized water (2 µl) were put on the surfaces of the specimen and after 10 seconds the contact angles were measured (n=10 measurements per material).

## Statistical analysis:

The SPSS version 27.0 (IBM Corp., Armonk, NY, USA) was used to perform statistical analyses. Shapiro-Wilk test was to be used to determine data normality. Multiple group comparisons were done with the help of one-way analysis of variance (ANOVA) and post-hoc test by Tukey. Time-dependent data which detected cytotoxicity were analyzed using repeated measures ANOVA. Paired comparisons were made with independent t -tests. The level of statistical significance was p<0.05. The results are given in the form of mean standard deviation.

## Results:

MTT assay results demonstrated that all tested materials maintained cell viability above 70% threshold at all-time points, indicating acceptable biocompatibility according to ISO 10993-5 criteria. Table 1 (see PDF) presents detailed cell viability percentages across different extraction periods. At 72 hours, Resin B demonstrated the highest cell viability among 3D printed materials (91.7 ± 3.8%), followed by Resin A (89.3 ± 4.2%) and Resin C (85.4 ± 5.1%). The control material exhibited viability of 93.2 ± 3.5%. Statistical analysis revealed significant differences between materials (p=0.012), with Resin C showing significantly lower viability compared to control (p=0.002) and Resin B (p=0.018), but not significantly different from Resin A (p=0.156). LDH cytotoxicity assay results corroborated MTT findings, with Resin C demonstrating the highest membrane damage (Table 2 - see PDF). Resin C exhibited significantly elevated LDH release (18.7 ± 2.9%) compared to control (9.2 ± 1.8%, p<0.001), Resin A (12.4 ± 2.1%, p=0.003) and Resin B (10.8 ± 1.9%, p<0.001). No significant differences were observed between Resin A, Resin B and control (p>0.05). Fluorescence microscopy examination of cell morphology revealed that HGFs exposed to Resin A, Resin B and control extracts maintained typical spindle-shaped morphology with well-organized cytoskeletal structures. Cells exposed to Resin C extracts showed slightly increased cellular rounding and reduced spreading area, though overall morphology remained largely preserved. Agar diffusion testing revealed minimal to absent inhibition zones for all tested materials against the three microorganisms (Table 3 - see PDF), indicating lack of significant antimicrobial activity in the materials themselves.

The microbial adhesion tests showed a significant difference between materials to colonize *S. mutans* (p<0.001). The lowest *S. mutans* adhesion value was observed with Resin B (2.3 ± 0.4 x 10^5^ CFU/ ml, p=0.002), which was significantly lower than control (4.1 ± 0.6 x 10^5^ CFU/ ml, p=0.001) and Resin C (3.6 +0.7 x 10^5^ CFU/ ml, p=0.002). The adhesion of Resin A was intermediate (3.1 +/-0.5 x 10^5^ CFU/ml), which was significantly less than control (p=0.012) but not significantly different than Resin B (p=0.087). In the case of *P. gingivalis*, there were slight differences (p=0.041) with Resin B showing the lowest adhesion (2.4 ± 0.5 x 10^5^ CFU/ml) and control showing the highest (3.5 ± 0.6 x 10^5^ CFU/ml) yet post-hoc comparisons showed no statistically significant pairwise differences. There were no significant differences between materials in *C. albicans* adhesion (p=0.062). Statistical significance difference of contact angles between materials was found to be statistically significant (p=0.003). Control material showed the greatest contact angle (75.9 + 4.5°), which is more hydrophobic material, then Resin B (72.5 + 3.8°), Resin A (68.3 + 4.2°) and Resin C (64.1 + 5.1°). The contact angle of resin C was considerably lower than that of control (p=0.002) and the other pairwise comparisons were not significant. The SEM analysis showed differences in surface topography. Control material showed highly smooth and homogeneous surfaces with little discontinuities. The surfaces of Resin A and Resin B were rather smooth and had small uneven spots and layer lines typical of 3D printing. Resin C presented more intense surface undulations and observable stratification of layers on large magnifications.

## Discussion:

This was an *in vitro* study on the biocompatibility, cytotoxicity and antimicrobial properties of three commercially available 3D printed aligner resins in contrast to conventional thermoformed material. The key results show that all the tested 3D printed materials had good biocompatibility profiles based on the ISO 10993-5 standards with slight differences in the cytotoxicity and surface-related microbial adhesion properties. The findings of the MTT cell viability assay showed that all the materials had a viability level above 70% threshold at all the time points, which meet the basic biocompatibility standards of intraoral devices [1]. Resin B, a urethane dimethacrylate-based 3D printed material showed the best cytotoxicity profile of any of the 3D printed materials with a cell viability (91.7) approaching the established polyurethane control (93.2). On the other hand, the hybrid composite Resin C exhibited relatively lower viability (85.4%) and higher LDH release, which implies a slightly higher cytotoxic property but still within the acceptable range [2]. The changes in cytotoxicity evident are probably due to the different chemical compositions of the resins, extent of polymerization and the amount of unreacted monomers. Methacrylate-based photopolymers have great mechanical properties but may not completely polymerize and thus, the components can be washed away, coming into contact with cellular membranes and metabolism [3]. The high LDH release of Resin C portrays a break of the membrane integrity and this may be explained by the presence of certain monomers, photoswitchable agents or additives in the hybrid formula [4]. Washing and post-curing procedures are considered post-processing protocols and have a strong impact on the biological safety of 3D printed materials, since they minimize the amount of uncured monomers (which remains) [5]. In this research, uniform post-processing according to the specifications of the manufacturers was used, but the differences between materials still existed, indicating that there would be inherent differences in compositions that would affect biocompatibility. The longer post-curing and washing regimens can further maximize the biological safety specifically on materials that are proving of marginal cytotoxicity [6]. The lack of notable antimicrobial activity using agar diffusion test of all materials demonstrates the fact that the current formulations of aligners do not have a natural antimicrobial substance. This observation is in line with the earlier studies of orthodontic polymers, which do not normally include antimicrobial additives which in turn may raise issues of biocompatibility, change in mechanical property and long term stability [7]. Nonetheless, the difference in microbial adhesion noted especially the lower colonization of *S. mutans* on Resin B indicates that the surface properties play a major role in bacterial adhesion. The adhesion of microbes is regulated through complicated interactions of the surface energy, hydrophobicity, roughness and chemical composition [8]. The reduced contact angle of a few of the 3D printed materials suggests greater hydrophilicity, which is typically associated with decreased bacterial adhesion of some species [9]. But because of layer-by-layer fabrication, surface roughness is created and this roughness could provide niches where bacteria can get established, canceling the advantage of hydrophilicity [10]. The comparatively low surface roughness of Resin B coupled with moderate hydrophilicity could be the causes of good adhesion resistance by *S. mutans*. These findings have many clinical consequences. To begin with, the acceptable biocompatibility of the 3D printed aligner materials can enable their safe clinical use, though the right material choice and post-processing guidelines are adopted [11]. Second, the small differences in cytotoxicity indicate that clinicians must pay attention to material-specific properties in the treatment of patients who are known to be affected by sensitivities or damaged oral tissues [12]. Third, the absence of antimicrobial properties underscores the ongoing need to educate patients on aligner hygiene, cleaning procedures and wear time to reduce the presence of microbes [13]. Future study areas should look into antimicrobial surface modification on 3D printed aligners, which may include addition of silver nanoparticles, quaternary ammonium compounds, or antimicrobial peptides, at the expense of biocompatibility [14]. Moreover, the intraoral condition simulators of the long-term aging studies should be conducted to determine the effects of mechanical stress, thermal cycling and exposure to oral fluids on biological properties during the treatment period. Prospective comparative studies of patient- reported outcome, soft tissue response and microbial profile of 3D printed and conventional aligners would offer informative real world evidence [15]. Limitations of the study are that the study was *in vitro* and could not completely model the complex oral environment with saliva, biofilm dynamics and mechanical forces. Although microbial testing on a single-species basis is a controlled microbial test, it is not a polymicrobial biofilm as seen in clinical settings. Also, it was only assessed in a single cell type (gingival fibroblasts); other appropriate cells like keratinocytes and immune cells should be incorporated in future studies in order to fully evaluate the biological responses.

## Conclusion:

3D printed aligner materials demonstrated biocompatibility comparable to conventional thermoformed aligners, supporting their safe use in orthodontic applications. Urethane dimethacrylate-based resins showed the most favorable biological response with minimal cytotoxicity. Incorporating antimicrobial surface modifications could further improve the clinical performance and hygiene of next- generation 3D printed aligners.
